# Microbial communities across a hillslope‐riparian transect shaped by proximity to the stream, groundwater table, and weathered bedrock

**DOI:** 10.1002/ece3.5254

**Published:** 2019-06-13

**Authors:** Adi Lavy, David Geller McGrath, Paula B. Matheus Carnevali, Jiamin Wan, Wenming Dong, Tetsu K. Tokunaga, Brian C. Thomas, Kenneth H. Williams, Susan S. Hubbard, Jillian F. Banfield

**Affiliations:** ^1^ Earth and Planetary Science University of California Berkeley California; ^2^ Earth and Environmental Sciences Lawrence Berkeley National Lab Berkeley California

**Keywords:** metabolism, metagenomics, microbiology, riparian, soil, watershed

## Abstract

Watersheds are important suppliers of freshwater for human societies. Within mountainous watersheds, microbial communities impact water chemistry and element fluxes as water from precipitation events discharge through soils and underlying weathered rock, yet there is limited information regarding the structure and function of these communities. Within the East River, CO watershed, we conducted a depth‐resolved, hillslope to riparian zone transect study to identify factors that control how microorganisms are distributed and their functions. Metagenomic and geochemical analyses indicate that distance from the East River and proximity to groundwater and underlying weathered shale strongly impact microbial community structure and metabolic potential. Riparian zone microbial communities are compositionally distinct, from the phylum down to the species level, from all hillslope communities. Bacteria from phyla lacking isolated representatives consistently increase in abundance with increasing depth, but only in the riparian zone saturated sediments we found Candidate Phyla Radiation bacteria. Riparian zone microbial communities are functionally differentiated from hillslope communities based on their capacities for carbon and nitrogen fixation and sulfate reduction. Selenium reduction is prominent at depth in weathered shale and saturated riparian zone sediments and could impact water quality. We anticipate that the drivers of community composition and metabolic potential identified throughout the studied transect will predict patterns across the larger watershed hillslope system.

## 1 INTRODUCTION

Soil microbial communities impact our environment by driving biogeochemical cycles from centimeter to global scales (Rousk & Bengtson, 2014; Schimel & Schaeffer, 2012). They expedite rock weathering (Gorbushina, 2007; Krumbein, 1988) recycle organic material in the subsurface, and facilitate the growth of vegetation by altering the availability of nutrients in the soil (Wardle et al., 2004). These changes influence soil nutritional status and productivity and plant survival and biotic interactions.

Mountains contribute the majority of water discharge in river basins (Viviroli, Weingartner, & Messerli, 2003) and were previously considered to be the origin of much of the world's water resources (Rodda, 1994). In recent years, studies have also addressed their contribution to subsurface carbon storage and carbon cycling (Chang et al., 2014; Hagedorn et al., 2010; Wan et al., 2018). These environments are comprised of a complex system of components, such as forests and meadows, floodplains, and glaciers. In turn, each of these accommodates various habitats including soil, bare rock, permafrost, and snow. Development of a predictive understanding of the behavior of such a heterogeneous and interconnected set of ecosystem compartments is an extremely complicated undertaking. Employing a scale‐adaptive approach in which different ecosystem compartments are considered as “systems within systems” could assist in disentangling the processes that shape overall mountain ecosystem function (Hubbard et al., 2018; Levin, 1992). A first step toward such a goal is to investigate structure and functioning within individual montane ecosystem compartments to provide a basis for future comparative studies and modeling efforts. In the long term, the “systems within systems” approach may better enable predictions accompanying natural or anthropogenic environmental perturbations.

Hillslope and floodplain compartments host the majority of soils in alpine and subalpine mountain ecosystems, and biogeochemical processes that occur there impact downstream ecosystems. Runoff and groundwater transport solutes along the elevation gradient and into aquifers, rivers, and lakes. Soils on hillslopes and in floodplains, and in general, harbor considerable microbial diversity (Donhauser & Frey, 2018; Frey et al., 2016; Rime et al., 2014). Most studies of microbial communities in mountainous soils have been concerned with the microbial community structure across different climate zones on the mountain slopes (Bardelli et al., 2017; Djukic, Zehetner, Mentler, & Gerzabek, 2010; Klimek et al., 2015; Xu et al., 2014; Zhang, Liang, He, & Zhang, 2013). However, most work has focused only on shallow soil, down to 20 cm (Bardelli et al., 2017; Yuan, Si, Wang, Luo, & Zhang, 2014; Zhang et al., 2013) and sometimes only the top 5 cm (Singh et al., 2014). The shallow layer of soil is profoundly affected by low temperatures that frequently drop below 0°C and snow cover that crucially limits biological, chemical, and physical processes, and thus microbial life (Zumsteg, Bååth, Stierli, Zeyer, & Frey, 2013). In contrast, the deeper soils and weathered rock in mountain ecosystems have been little studied. While affected by events taking place in shallow layers, the microbial communities there are probably also influenced by moisture gradients and the geochemistry of the underlying bedrock (Tytgat et al., 2016).

The East River headwaters catchment is a mountainous, high‐elevation watershed, dominated by the Cretaceous Mancos Shale Formation, with carbonate and pyrite contents of roughly 20% and 1%, respectively (Morrison, Goodknight, Tigar, Bush, & Gil, 2012). The watershed has a mean annual temperature of ~0°C, with average minimum and maximum temperatures of −9.2°C and 9.8°C, respectively. The watershed receives ~600 mm of precipitation per year, the bulk of which falls as snow, and is representative of many other headwaters systems within the upper Colorado River Basin (Hubbard et al., 2018; Pribulick et al., 2016).

The present research focused on a lower montane hillslope through floodplain transect located within the East River, CO watershed, which is the focus of the Lawrence Berkeley National Laboratory‐led Watershed Function Project. The intensively studied site investigated in the current study is referred to as PLM (Pump House Lower Montane). The Watershed Function Project builds upon a scale‐adaptive investigation, which focuses on different spatial and temporal scales within the East River watershed, explores how mountainous watersheds retain and release water, nutrients, carbon, and metals downgradient (Hubbard et al., 2018). The current study aims to lay the groundwork for the scale‐adaptive, system within systems approach by identifying ecological niches of interest that would later be tested in a bottom‐up approach across the watershed. We hypothesize that microbial community composition and metabolic potential is similar among sites along an altitudinal transect down the hillslope and that hillslope communities differ from those of the floodplain riparian zone. Furthermore, we hypothesize that proximity to shale and groundwater will affect the composition and functionality of microbial communities, differentiating hillslope communities from other watershed microbial consortia.

## 2 METHODS

### 2.1 Site description and sample collection

**Figure 1:**
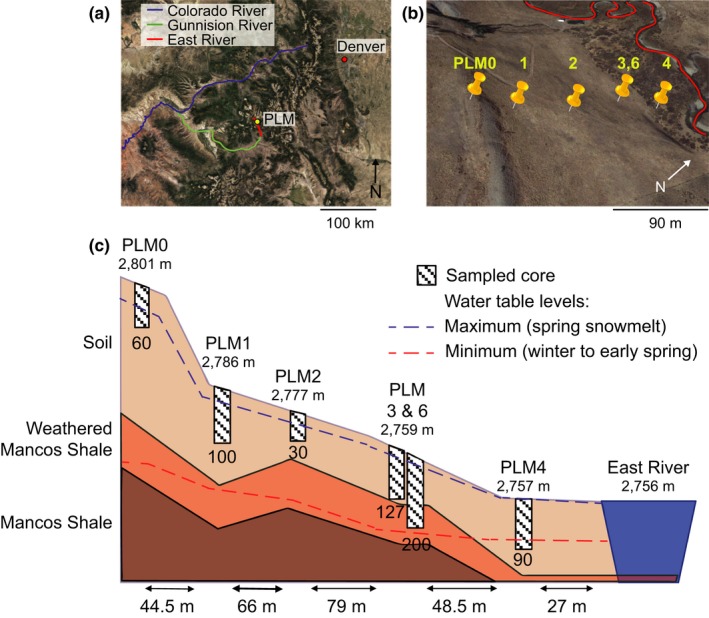
East River Watershed hillslope‐riparian zone transect sampling sites. (a) The location of East River PLM intensive study site. (b) Five PLM sites are located across a hillslope transect. PLM0 is the highest point of the transect, and PLM4 is located in the floodplain. (c) Schematic representation of the sampling sites. Elevation of the surface, given in meters above sea level, appears below the name of the sampling site. Maximum depth at each sampling site is specified below the depiction of the sampled core in centimeters. Horizontal distances between sites are given at the bottom of the illustration. Maximum and minimum water levels are depicted by dashed blue and red lines, respectively. The PLM6 site was initially drilled for another study, 5 m from PLM3 but at the same elevation. A full view of the East River watershed is given in Figure A1

**Figure A1:**
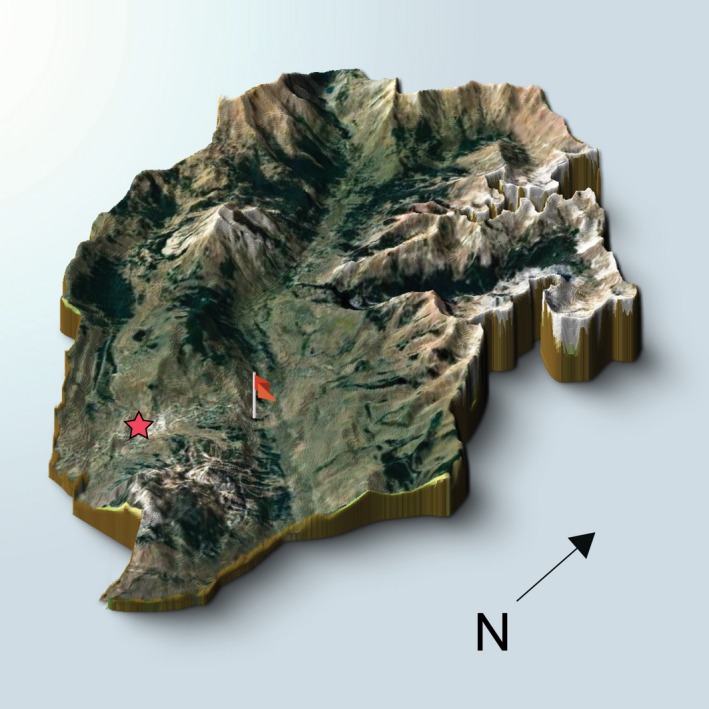
Illustration of the East River watershed. Star and flag indicate the town of Crested‐Butte and the location of the Pumphouse Lower Montane (PLM) sampling site, respectively

The PLM intensive study site is located on the northeast facing slope of the East River valley near Crested Butte, Colorado, USA (38°55′12.56″N, 106°56′55.39″W) (Figures 1 and A1). Exact locations were determined at an accuracy of 0.5 m with a Trimble Geo 7X GPS. All samples were collected during three days in September 2016 from meadow sites before any intensive research activities were performed. The ground surface at each site was cleared of vegetation with a hand trawler prior to sampling. Samples were collected with a manual corer lined with 7.6 cm tall and 15.2 cm diameter bleached sterile plastic liners. Five soil profile sampling sites abbreviated PLM0, PLM1, PLM2, PLM3, and PLM4 were chosen along a 230 m hillslope transect. The profiles terminated at depth in the unsaturated zone, with the exception of PLM4, which extended below the water table. The base of PLM3 and PLM6 profiles is located near or within the weathered Mancos Shale bedrock, while the base of PLM0 was located >1 m above the weathered bedrock. PLM0 is at the top of the hill and PLM4 on the East River floodplain, 2,804 m and 2,757 m above sea level, respectively (Figure 1). One full core was taken at each sampling depth, and the soil in between sampling depths was removed with an auger. An additional site, PLM6, was sampled by drilling and provided access to weathered shale. Samples at PLM6 were taken from a split‐spoon, dry drilled core. In total, 20 samples were collected as follows: PLM0—5, 30, 60 cm; PLM1—5, 30, 60, 100 cm; PLM2—5, 30 cm; PLM3—5, 30, 60, 127 cm; PLM6—50, 170, 200 cm; PLM4—5, 32, 65, 90 cm.

Immediately after extraction, a sample from each site and depth collected within an individual sterile plastic liner was placed in a sterile Whirl‐Pak bag and manually homogenized. Aliquots of 5 g of soil from each bag were placed in 10 ml of LifeGuard Soil Preservation Solution for RNA and DNA co‐extraction, whereas the rest of the sample was used for DNA extraction. Care was taken to avoid roots and small rocks. Samples in sterile Whirl‐Pak bags and preservation solution were placed in a chilled cooler until processing at the Rocky Mountain Biological Laboratory (RMBL) later that day. In the laboratory, roots and small rocks were removed from sampling bags, and three 10 g subsamples were weighted from each sample and placed in a −80°C freezer. Samples were shipped overnight on dry ice to University of California, Berkeley for DNA and RNA extractions.

Particle size analyses of samples were conducted according standard methods (Gee & Or, 2002). Geochemical measurements were made at the Earth and Environmental Sciences department's Aqueous Geochemistry Laboratory. Water soluble cation–anion composition was measured by water extraction (1:1 soil:DIW mass ratio) and ICP‐MS. Total inorganic carbon (TIC) and total organic carbon (TOC) in soil samples were determined using a Shimadzu TOC‐VCSH total inorganic and organic carbon analyzer combined with a solid sample combustion unit of SSM‐5000A. Total nitrogen (TN) was analyzed using a Shimadzu Total Nitrogen Module (TNM‐1) combined with the TOC‐VCSH analyzer. pH was measured with an uncertainty of ±0.05. For TIC/TOC and IC the uncertainty is <3% and <5%, respectively. All geochemical measurements for samples taken at PLM6, nitrate concentration for the sample from PLM0 30 cm, and sulfate concentrations for samples PLM0 40 cm, PLM1 60 cm, PLM1 90 cm, PLM2 5 cm, and PLM2 30 cm are not available.

### 2.2 DNA extraction and sequencing

DNA was extracted from 10 g of soil with DNeasy PowerMax Soil Kit in two batches of 5 g each which were combined during the cleaning step. Extraction process followed the manufacturer's protocol with the following modifications: Soil was vortexed at maximum speed for an additional 3 min in the sodium dodecyl sulfate reagent and then incubated for 30 min at 60°C, with intermittent shaking in place of extended bead beating, as established by Hug et al. (2015). For DNA precipitation, sodium acetate (1:10 v/v) and isopropanol (1:1 v/v) were added and samples were incubated overnight (−20°C). Following incubation, DNA was pelleted by centrifugation (15,300 g, 15 min, 4°C), washed with cold ethanol, and suspended in ddH_2_O. DNA was further cleaned with DNeasy PowerClean Pro Clean Up Kit following the manufacturer's protocol.

DNA was also co‐extracted with RNA from 5 g of soil using RNeasy PowerSoil Total RNA Kit and Phenol:Chloroform:Isoamyl Alcohol 25:24:1 saturated with 10 mM Tris (final pH 8.0) and 1 mM EDTA. RNeasy PowerSoil DNA Elution Kit was used to collect DNA which was further cleaned using DNeasy PowerClean Pro Clean Up Kit. The co‐extraction and cleaning steps were conducted according to the manufacturer's protocol. While RNA was extracted for the purpose of another study, using co‐extraction as a second extraction method was expected to improve the detection of the total diversity of microbes in the sample (İnceoǧlu, Hoogwout, Hill, & Elsas, 2010). Overall, two DNA samples were produced from each sampling, one from DNA extraction and the second from the DNA that was co‐extracted along with RNA. A third DNA sample was extracted from the 90 cm deep PLM4 sample; thus, a total of 41 DNA samples were used for further analysis.

Metagenomic libraries were prepared at the Joint Genome Institute (JGI) after validating concentrations and DNA integrity using Qubit (Thermo Fisher Scientific) and gel electrophoresis, respectively. Libraries were prepared using NEB's Ultra DNA Library Prep kit (New England Biolabs) for Illumina with Ampure XP bead selection aimed to give fragments of 500 base‐pair (bp) according to the manufacturer's protocol. The library was sequenced at JGI using an Illumina Hiseq 2500, resulting in paired‐end, 150 bp sequences.

### 2.3 Bioinformatic analyses

Raw reads processing followed protocols described elsewhere (Hernsdorf et al., 2017). Briefly, reads were trimmed based on quality scores with Sickle (Joshi & Fass, 2011) and assembly was accomplished with IDBA‐UD v1.1.1 (Peng, Leung, Yiu, & Chin, 2012) using kmer size range of 40–140. Only assembled scaffolds with >1 kbp were included in downstream analysis. Open reading frames were identified by Prodigal v2.6.3 (Hyatt et al., 2010) using the metagenomic setting.

Microbial community structure was assessed according to the abundance of the ribosomal protein S3 (*rpS3*) marker gene (Brown et al., 2015) by modifying the method described by Anantharaman et al. (2016). Archaeal, eukaryotic, and bacterial *rpS3* protein sequences were identified using Hidden Markov Models (HMM) (Finn et al., 2015). Ten *rpS3* reference sequences which compose TIGRFam's TIG01009 model were added to the protein sequences that were identified by HMMs and aligned with MAFFT (Katoh & Standley, 2013). Positions within the alignment with >95% gaps were removed, leaving 206 amino acids in the longest, nonreference sequence. Sequences that had less than 103 nongap positions (50% of overall nongap positions) were removed from the analysis. This step ensured that only positions that are truly related to the sequence of *rpS3* were included in downstream analysis.

The amino acid sequences were clustered with the cluster_fast algorithm from USEARCH software (Edgar, 2010) at a 99% similarity threshold, and the following settings: query_cov = 1, target_cov = 0.5, and both max_accept and max_reject set to 0. Scaffolds of DNA sequences that matched the clusters’ open reading frames were retrieved from the metagenomes. Average coverage was used as a proxy for relative abundance of different sequence types. In this analysis, the scaffolds were trimmed to include 2 kbp flanking the *rpS3* gene. If the scaffold spanned less than 2 kbp on both sides, then the entire scaffold was kept, with a minimal length of 1 kbp. The relative abundance of each trimmed scaffold was determined by mapping the reads from each sample to each trimmed scaffold with bowtie2 (Langmead & Salzberg, 2012). The average coverage and breadth of coverage of each scaffold in each sample was then calculated (Olm et al., 2017). Each scaffold is considered to be present in at least one sample (at minimum, the sample from which it was originally assembled) but could be falsely identified in other samples due to a low breadth cutoff (i.e., false positive). Therefore, we implemented a breadth cutoff of 0.72 based on iterating breadth cutoffs of 0.1 to 1, to find the lowest breadth cutoff that would retain the same number of clusters as went into the analysis. The abundance of organisms at each site was calculated as the average abundance for the two samples (or three in the case of PLM4 at 90 cm) extracted from that site.

Genes involved in carbon, nitrogen, and sulfur metabolism were identified using 86 previously published HMM models (Anantharaman et al., 2016), and KEGG KOfam database (Aramaki et al., 2019) (Table A1). Additionally, *srdA* which encodes for a membrane‐bound catalytic subunit of selenate reductase was detected with a custom HMM model. The model was constructed by aligning 20 amino acid sequences, 934–1222 aa long, determined to be included in the *srdA* specific clade (Harel, Häggblom, Falkowski, & Yee, 2016). All matches from HMM search for *srdA* were aligned, and a threshold was decided upon according to their clustering in a phylogenetic tree. Score cutoffs for custom made and PFAM HMMs were manually validated and adjusted by aligning the HMM search results, plotting a phylogenetic tree using FastTree v2.1.9 (Price, Dehal, & Arkin, 2010), and interrogating clades with NCBI's BLASTP (Boratyn et al., 2013) against nr database. The abundance of each gene was determined by mapping the reads from each sample to each scaffold and calculating the average coverage using the same breadth cutoff as before.

### 2.4 Taxonomy and phylogeny

The longest amino acid sequence from each *rpS3* protein sequence cluster was selected as a representative and was compared to a database of *rpS3* protein sequences (Hug, Baker, et al., 2016; Hug, Thomas, et al., 2016) using the UBLAST function in USEARCH (Edgar, 2010). Results were filtered to include only the top hits with *e*‐values < 1*e*−5. While each cluster roughly correlates with a species, not all clusters could be taxonomically identified to that level. Therefore, further investigation relied on phylogenetic distance, which enables a high‐resolution analysis. A phylogenetic tree was created by aligning only the representative amino acid sequences using MAFFT with an automated strategy (Katoh & Standley, 2013) and trimming noninformative positions. A maximum‐likelihood tree was constructed on CIPRES (Miller, Pfeiffer, & Schwartz, 2010) with RAxML (Stamatakis, 2014), using the LG substitution model and bootstrapping, allowing the software to halt bootstrapping once it reached a consensus. The Eukaryote domain branch was set as root, and the tree was manually inspected for errors. The phylogenetic tree along with *rpS3* gene abundance heatmap were visualized with iTol v4.2.3 (Letunic & Bork, 2016).

### 2.5 Statistics

Statistical analysis was conduct in R v3.4.3 (R Development Core Team, 2012) and Rstudio v1.1.423 (Rstudio Team, 2015). Abundance plots, ordinations and UniFrac calculations were conducted with Phyloseq v1.22.3 (McMurdie & Holmes, 2013). The abundance of each *rpS3* cluster was corrected for uneven sequencing depth across samples by multiplying the coverage value for each sample by a factor calculated as the ratio of the number of bp in the largest sample divided by the number of bp in that sample.

Factor selection of soil chemistry was carried with BIOENV (Clarke & Ainsworth, 1993) as implemented in the bio.env function from Vegan v2.4.6 (Oksanen et al.., 2018), with a Euclidean distance method and Bray–Curtis matrix. The exhaustive search for correlation between community dissimilarities and environmental distances requires extremely long time. Therefore, dissimilarities were partialled out when inspecting variables as recommended by the bioenv user's manual (Oksanen et al., 2018). The results were evaluated with Pearson's correlation. The significance of the results was validated with Mantel test also using Pearson's correlation. Maps were retrieved from Google maps database using Google Earth v7.3.2.

## 3 RESULTS

**Figure A2:**
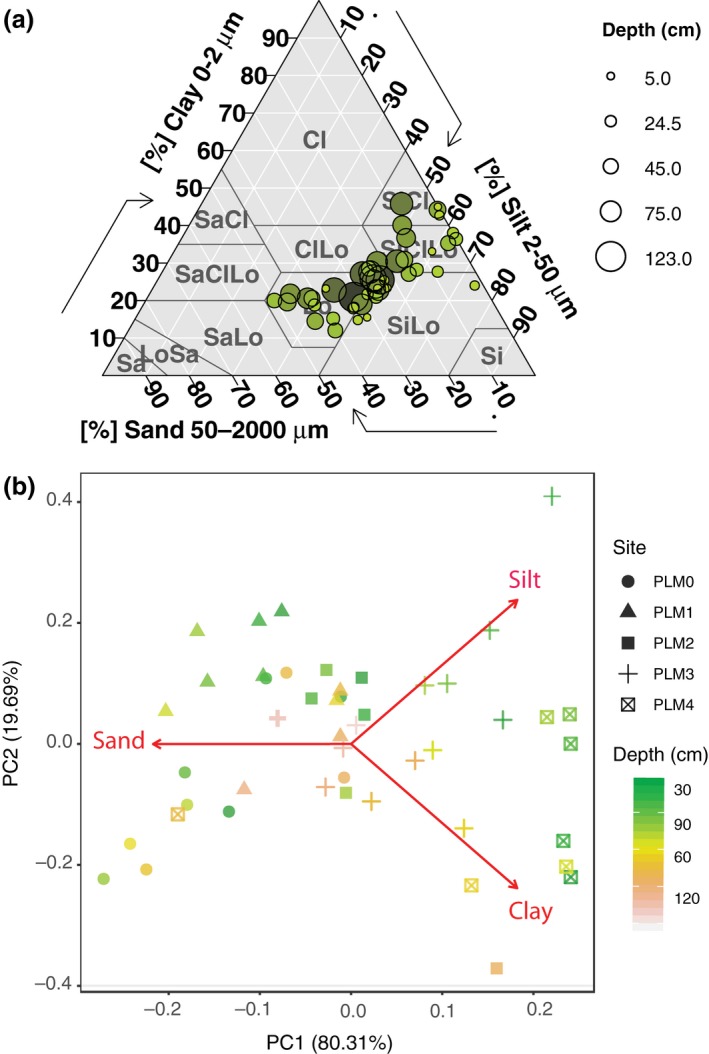
Soil texture. All soils are categorized as silty‐loam (A) with the greatest variability being 20‐60% sand content. PLM0 and PLM1 are found to contain more sand than in PLM3 and PLM4 (B) and sand content is correlated with the PC1 which explains almost 80% of the variability between the samples

**Figure A3:**
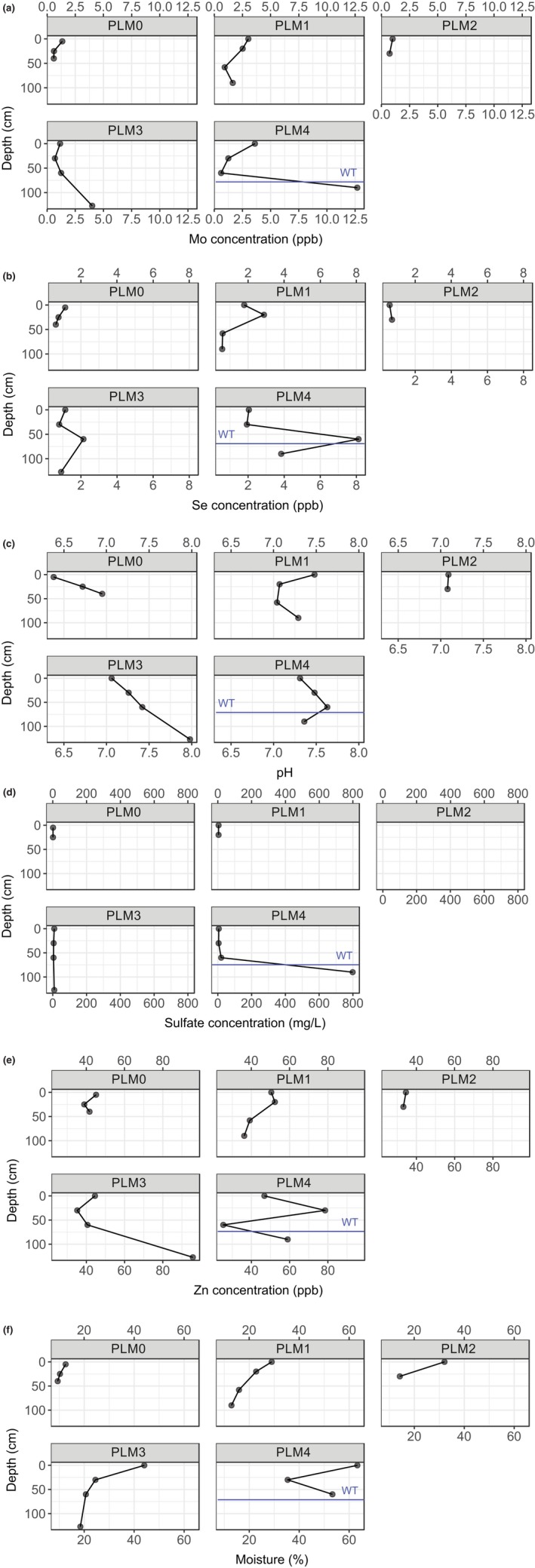
Geochemistry measurements of elements that showed a correlation to microbial community structure. Data is missing for PLM6. Sulfate measurements are missing for PLM0 40 cm, PLM1 60 cm and 90 cm, PLM2 5 cm and 30 cm

For the hillslope samples analyzed, the soils are loamy to silty loam (Figure A2 and Table A3). Shallow samples from PLM0 and PLM1 have higher sand content than downslope PLM3 and PLM4 samples, which have higher content of clay and silt, potentially as a result of downslope fining of transported sediments. Soil moisture increases with proximity to the East River, but decreases with depth (Figure A3 and Table A4). An exception to this is at the floodplain, where moisture increases close to the water table (72 cm below the ground surface at the time of sampling). The hillslope meadow is dotted with smooth brome (*Bromus inermis*) and lupines (*Lupine* sp.); however, neither occurred within a 50 cm radius of the sampling sites (qualitative assessment on site). In contrast, the floodplain is dominated by willows and sedges that are not present on the hillslope. Gopher activity increases downslope, but does not occur at the floodplain location (W. Brown, personal communication, February 2018).

**Figure 2:**
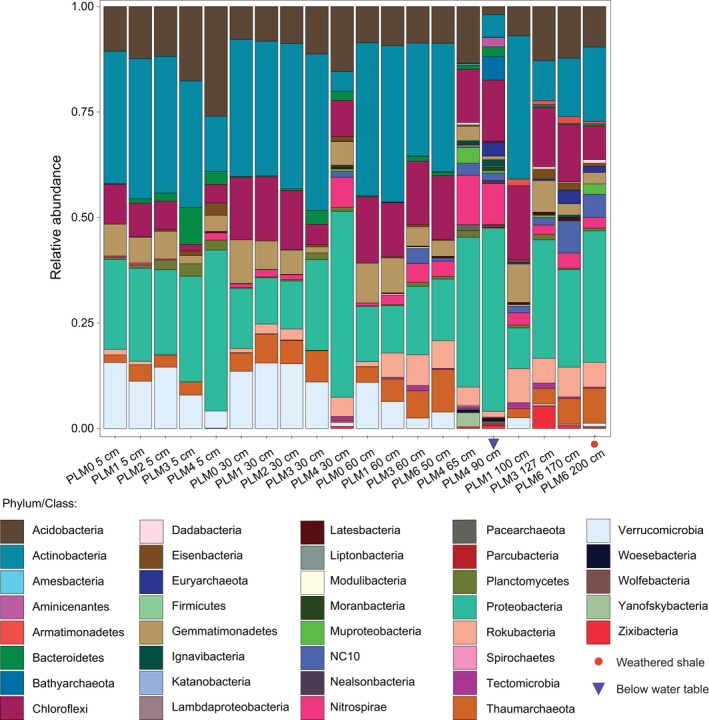
Relative abundances of phyla. Results show that Verrucomicrobia decrease in abundance with increasing depth and proximity to the floodplain site PLM4; Rokubacteria, on the other hand, show the opposite pattern

Assembling reads from 41 samples, comprising 610 Gbp of sequence data, resulted in 6.5 million scaffolds longer than 1 kbp (Table A2). On average, 27.8% (±11) of the reads could be mapped back to these scaffolds. This is an expected result given huge diversity in soil and the near flat nature of most of the rank abundance curve. The unassembled reads likely derive from the background of rare organisms in soil. Encoded on the assembled scaffolds, 3,536 *rpS3* amino acid sequences were identified and clustered into 1,660 clusters (at 99% identity), representing 37 microbial phyla. In general, the microbial communities are dominated by bacteria (relative abundance 0.95 ± 0.03 *SD*). The most abundant phyla across all samples are Acidobacteria, Actinobacteria, Chloroflexi, and Proteobacteria, but their relative abundances vary considerably across samples and depths (Figure 2). Species of Verrucomicrobia and Gemmatimonadetes are abundant at sites high on the hillslope, but while Verrucomicrobia species abundance decreases with proximity to the river (Pearson's *r* = −0.707, *p*‐value < 0.001), the abundance of Gemmatimonadetes is correlated with both proximity to the river (Pearson's *r* = −0.652, *p*‐value < 0.001) and soil depth (Pearson's *r* = −0.568, *p*‐value < 0.001).

**Figure 3:**
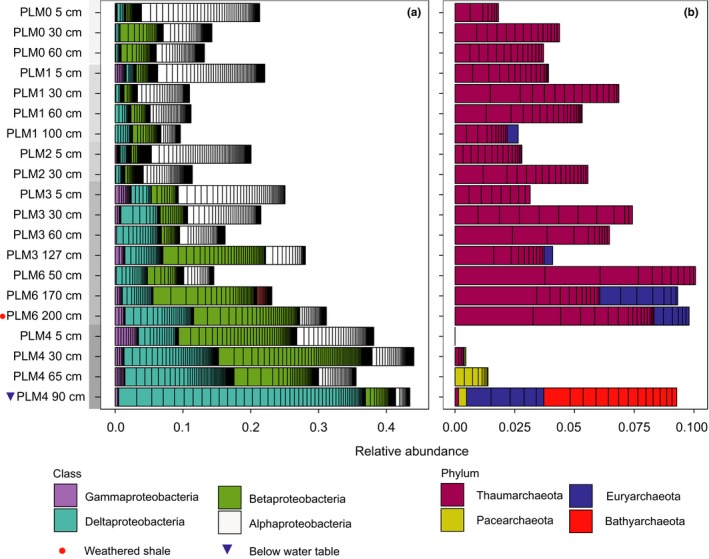
Relative abundances of proteobacterial classes (a) and archaeal phyla (b) clusters across the sampling sites. Within bars of the same color, black lines separate distinct organisms. Samples are ordered from the top to the bottom of the hillslope transect. Within each site, samples are ordered by depth

**Figure A4:**
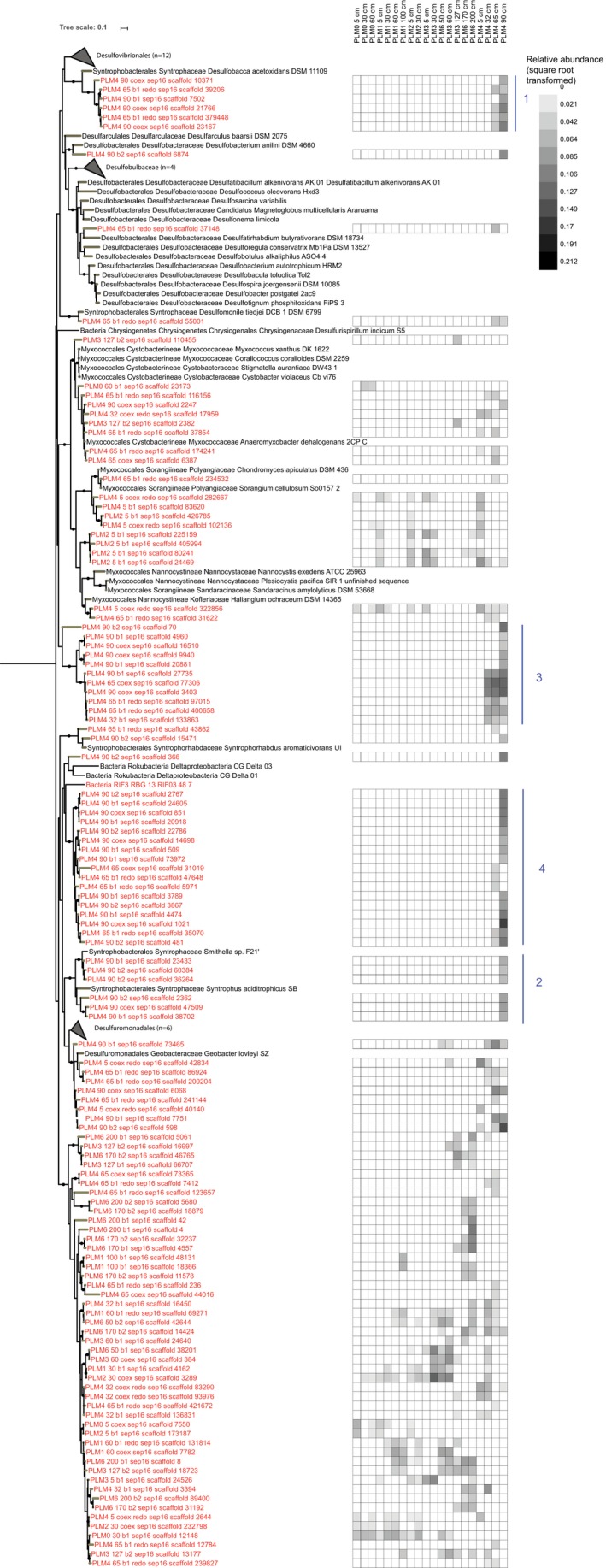
A Maximum‐Likelihood phylogenetic tree of rpS3 clusters classified as Deltaproteobacteria. Black circles mark branch support greater than 0.8. Grey scale bar was calculated with the square root of relative abundance of each cluster. Clades of interest are marked 1 through 5

Proteobacteria species comprise 22.7% (±10.8 *SD*) of all microbial abundance. This dominance increases systematically with distance down the hillslope, largely irrespective of the sampling depth (Figures 2 and 3a). Gammaproteobacteria species are almost undetectable in communities higher on the hillslope, whereas alphaproteobacterial species are prevalent at all sites (Figure 3a). Deltaproteobacteria species increase in abundance with increasing proximity to the floodplain and also with increasing proximity to the water table, with the highest representation observed in samples from below the water table. Distinct Deltaproteobacteria species are found in samples close to the water table (*Desulfobacca acetoxidans* in clade 1, and *Geobacter* spp. and *Desulfuromonas* sp. in clades 3 and 4, see Figure A4). Some distinct species (clade 2 in Figure A4) occur only below the water table (Syntrophaceae, Figure A4, clade 2). Thaumarchaeota related to *Nitrososphaera* sp. are the dominant archaea at every location other than at the floodplain (Figure 3b). At the floodplain site (PLM4), Pacearchaeota are present in soil samples close to, although above the water table whereas Bathyarchaeota and Euryarchaeota are present in samples below the water table.

**Figure 4:**
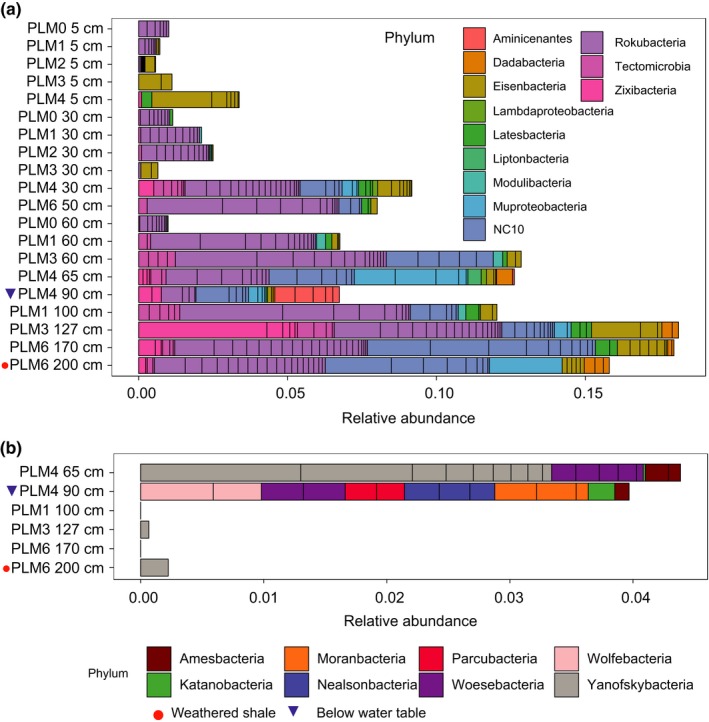
Abundances of Candidate phyla (CP) and Candidate Phyla Radiation (CPR) bacterial clusters at hillslope sites. (a) Abundance of bacteria from CP other than CPR phyla. (b) Abundance of bacteria from CPR phyla. Samples are ordered by depth and within any specific depth, from top to bottom of the hillslope transect. CPR phyla were not detected in samples other than the six depicted in this figure

Out of the 37 microbial phyla that were identified, 20 are candidate phyla (CP) (i.e., phyla that lack an isolated representative). Of the CP, eight are part of the Candidate Phyla Radiation of Bacteria (CPR) (Figure 4). Members of CP are present at all sites along the hillslope transect, but their detection is positively correlated with depth of sampling (Pearson's *r* = 0.851, *p*‐value < 0.0001) (Figure 4a). Moreover, depth could be used as a predictor for the abundance of CP as a linear regression has an *r* ^2^ = 0.66 and slope = 5.07 (*p*‐value < 0.0001). Interestingly, CPR bacteria are almost exclusively found at the floodplain site and only just above (7 cm above the water table) and within groundwater‐saturated sediment (Figure 4b). Although sampling sites above and below the water table are close spatially and may experience similar conditions when groundwater level fluctuate, they harbor bacteria from completely different CPR phyla.

**Figure 5:**
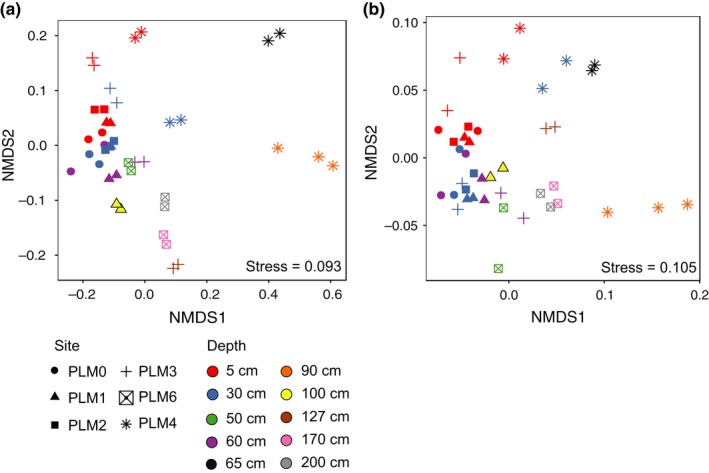
Samples cluster based on proximity to weathered shale and groundwater‐saturated soil. (a) NMDS based on unweighted UniFrac distance computed using maximum‐likelihood phylogenetic tree. (b) NMDS based on weighted UniFrac distances computed using maximum‐likelihood phylogenetic tree and abundance of each taxon. Confidence ellipses (95% interval) are shown in Figure A4

**Figure A5:**
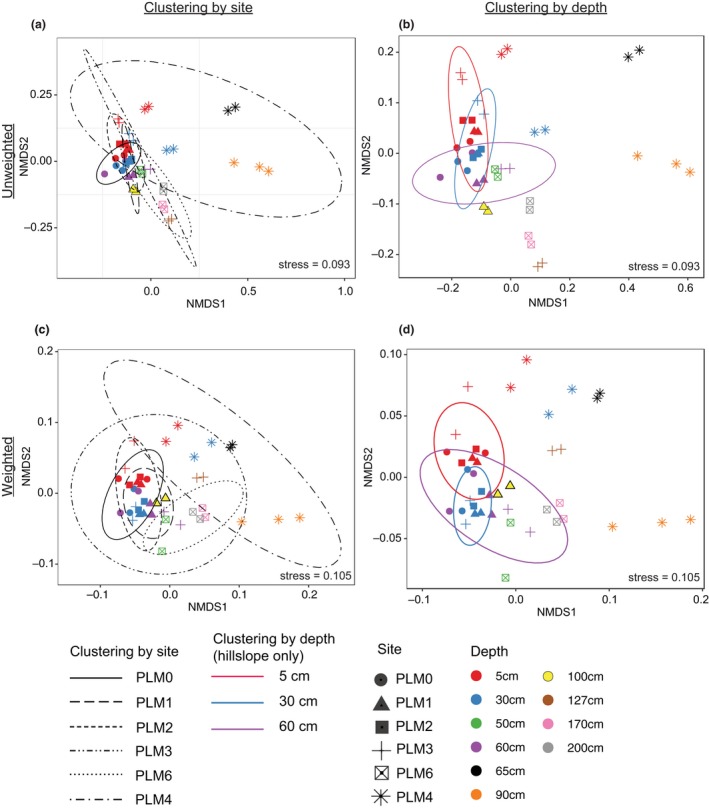
Samples cluster based on depth and distance from river. (A) and (B) NMDS based on unweighted UniFrac distance computed using Maximum‐Likelihood phylogenetic tree. Ellipses mark 95% confidence interval for samples grouped by site (A) or depth (B). Similar analysis by weighted UniFrac distances is shown in (C) and (D) where ellipses mark 95% confidence interval for samples grouped by site (C) or depth (D). Confidence ellipses were not calculated depths of 50 cm, 90 cm, 100 cm, 127 cm, 170 cm and 200 cm as there were not enough data points to conduct the statistic calculation. For confidence ellipses in (B) and (D) hillslope sites were considered separately from floodplain sites due to the their apparent sepration on the NMDS plot

We investigated how distance from groundwater and weathered shale impact microbial community structure. Unweighted UniFrac‐based PCoA ordination, that allows addressing phylogenetic distance without assigning taxonomic levels, reveals that soils sampled at depths of 5 cm and 30 cm from all field sites group together (Figures 5a and A5a and b). However, the weighted UniFrac PCoA analysis (considering organism abundances) differentiates these 5 cm from 30 cm soil samples. Considering distance from the river while suppressing information describing depth below ground surface, these analyses also differentiate samples taken at PLM4 from those taken at PLM0, PLM1, and PLM2 but not from PLM3, which is closer to the floodplain. Lastly, weighted UniFrac separates samples from PLM4 from above and below the water table (Figures 5b and A5c and d). Thus, for soils that contain similar types of organisms, sampling depth and proximity to weathered rock shift organism abundance relative levels. Overall, distance from groundwater at the floodplain site and weathered shale at the hillslope sites seem to be dominant factors in determining the microbial community structure across the hillslope.

**Figure 6:**
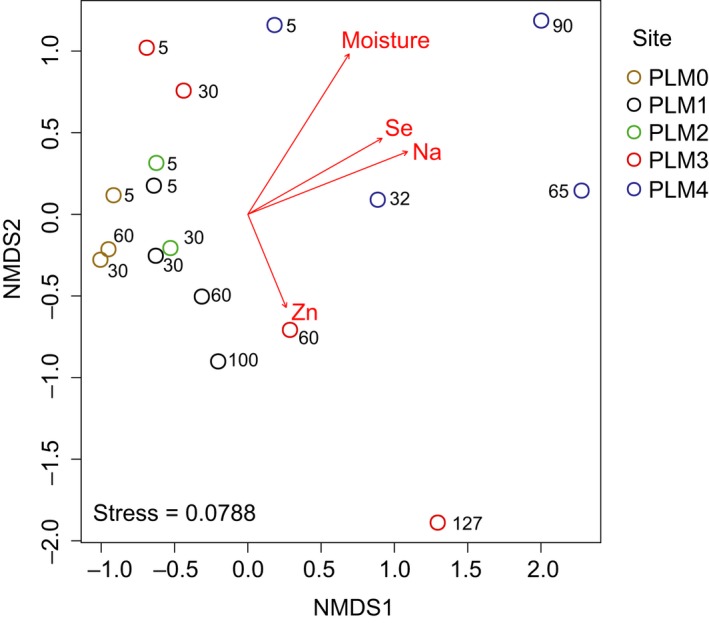
NMDS ordination of microbial communities and correlated geochemical factors. Spearman correlation was tested using Bray–Curtis distances and Euclidean distance matrix. Out of 40 geochemical measurements (Table A4) only soil moisture, Se, Na, and Zn were correlated with microbial community composition (*r* = 0.751, *p*‐value = 0.001). Stress = 0.0788. Numbers in figure are depth in cm. Raw values are provided in Table A4

Forty geochemical factors were assessed in order to elucidate the factors that shape community structure in the soil profile sites. The combination of soil moisture and concentrations of Na, Se, and Zn were correlated to microbial community structure (*r* = 0.751) (Figure 6). The results were validated with Mantel test (Pearson's *r* = 0.751, *p*‐value = 0.001, 999 permutations). Selenium had the highest concentration in samples taken above the water table, (PLM4 65 cm, 8.119 ± 0.235 ppb) whereas zinc concentrations were the highest in samples closest to weathered shale (PLM3 127 cm, 95.694 ± 0.915 ppb), which also had the highest acidity (pH = 7.98) (Table A4). Sodium (Na) concentrations were the highest in samples taken from below the water table (PLM4 90 cm, 9,178 ppb).

**Figure 7:**
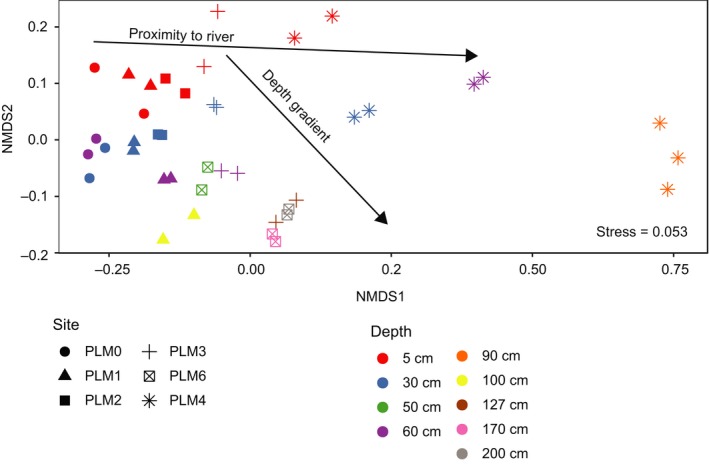
Abundance of key metabolic enzymes cluster samples according to depth and proximity to river. An NMDS of key metabolic genes generated using 78 HMMs of carbon, nitrogen, sulfur, and selenium metabolic enzymes

**Figure A6:**
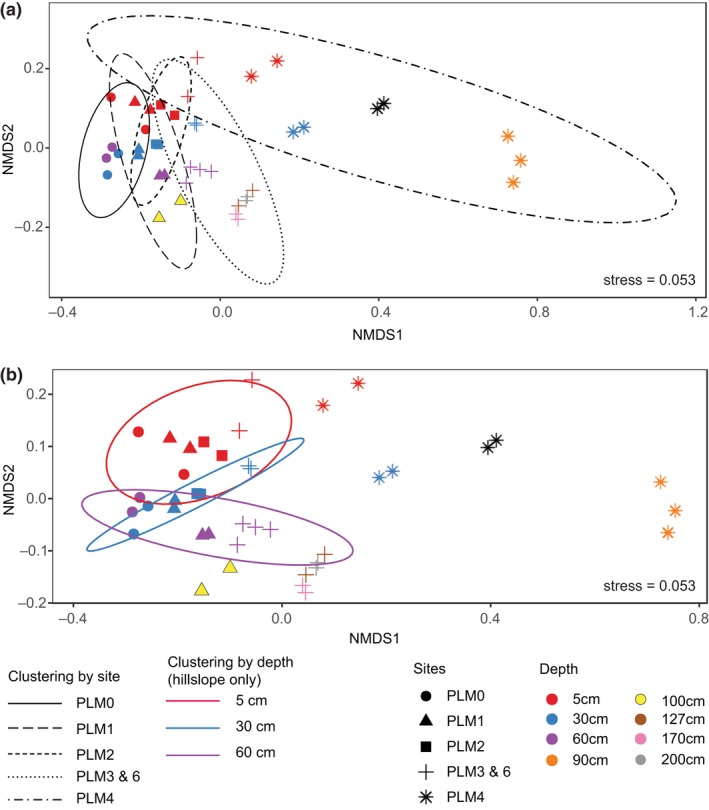
NMDS of samples according to abundance of key metabolic enzymes. A. NMDS of key metabolic genes generated using 79 HMMs of carbon, nitrogen, sulfur and selenium metabolic enzymes. Ellipses mark 95% confidence interval for samples grouped by site (A) or depth (B). Confidence ellipses were not calculated depths of 50 cm, 90 cm, 100 cm, 127 cm, 170 cm and 200 cm as there were not enough data points to conduct the statistic calculation. For confidence ellipses in (B) hillslope sites were considered separately from floodplain sites due to their apparent separation on the NMDS plot

Metabolic potential, as depicted by detected genes, differentiates locations along the hillslope to floodplain transect. Out of 87 Hidden Markov Models (HMMs), 78 were found to exceed our detection threshold (see Section 2). An NMDS of gene abundances reveals a clear depth gradient in samples taken from the floodplain site (Figures 7 and A6). A depth‐dependent trend in overall metabolic potential is also observed along the hillslope. In addition, gradient in overall metabolic potential correlates with elevation (i.e., position on the hillslope).

**Figure A7:**
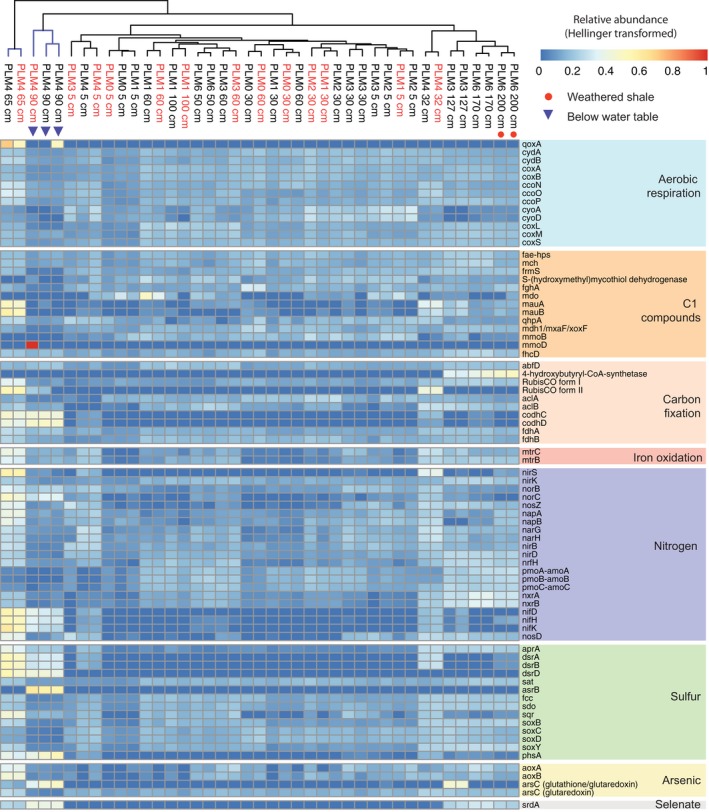
Spatial abundance of genes central to metabolic pathways. Samples from the floodplain (blue colored clade) are distinct from samples from across the hillslope (black colored clade), particularly with respect to carbon fixation and selenate reduction. Sample names in red denote DNA samples that were co‐extracted with RNA (see Section 2). The sources of HMMs their description and detection cutoffs are given in Table A1

The patterns identified in the NMDS are driven in part by genes encoding enzymes involved in N_2 _fixation (nifDHK), denitrification (*norBC* and *nosZ*), and the Wood–Ljungdahl carbon fixation pathway (*codhC* and *codhD*) (Figure A7). The *dsrA* and *dsrB* genes that encode reversible dissimilatory sulfite reductase are found in groundwater‐saturated saprolite samples PLM4 90 cm, in samples taken 10 cm above groundwater (PLM4 65 cm), and also present in samples collected at 5 cm depth. However, *dsrD* which is present only in samples from below groundwater and in samples taken 10 cm above it indicates that *dsrA* and *dsrB* are potentially responsible for sulfite reduction at these locations. Sequences of *asrB* which encodes for anaerobic sulfite reductase B were found exclusively in samples from groundwater saprolite (PLM4 90 cm). Also enriched in samples from below the water table is the catalytic subunit of thiosulfate reductase *phsA,* which catalyzes the reduction of thiosulfate to sulfite and hydrogen sulfide. Selenate reductase encoded by the gene *srdA,* which is associated with selenate respiration, is enriched in samples from below compared to above the water table and weathered shale compared to soil. The abundance of *srdA* was found to be correlated to selenium concentration (Pearson's *r* = 0.52, *p*‐value = 0.0325). Unfortunately, selenium measurements for PLM3 127 cm as well as PLM6 170 cm and 200 cm, where *srdA* abundance is the highest, were not available. These samples were taken from fractured shale which is rich with selenium, and therefore, it is assumed that adding these measurements will result in a stronger positive correlation.

## 4 DISCUSSION

We integrated metagenomics and soil chemical analyses to investigate how microbial community structure and metabolic potential vary within the subsurface across a transect from high on an East River hillslope to its adjoining floodplain. Our analyses indicate that communities are differentiated according to depth and proximity to weathered shale and groundwater, and that microbial communities of the floodplain soils and sediments differ substantially from those collected along the hillslope.

Notably, the abundance of species of Archaea, Proteobacteria and CPR bacteria have distinct spatial patterns. Thaumarchaeota, the dominant archaeal taxon in soils (Bates et al., 2011), are typically aerobic ammonium oxidizers that can drive nitrification (Colman, 2017). They were detected at every depth sampled across the hillslope, as found in hillslope soil pits in Colorado by Eilers, Debenport, Anderson, and Fierer (2012). The absence of Thaumarchaeota at the floodplain may be explained by extended periods of water saturation. Low redox conditions, inferred based on abundant genes involved in sulfate and selenate reduction, apparently selected instead for Bathyarchaeota and Euryarchaeota. The decrease in relative abundance of Alphaproteobacteria and Gammaproteobacteria with depth has been previously described in soil profiles from upper montane forest east of Boulder, CO, USA (Eilers et al., 2012). However, while the relative abundance of Betaproteobacteria was reported to decline with depth in the Boulder site, it mostly increased with depth at the hillslope. A similar pattern of increased relative abundance is observed in Deltaproteobacteria. It could be that the proximity to sulfate and nitrate rich Mancos shale bedrock supports the increased abundance of these organisms.

Bacteria from CP increase in abundance with depth throughout PLM sites. They may have eluded prior cultivation studies due to their low abundances in more commonly sampled shallow soils. However, CPR bacteria, which elude most cultivation efforts (Solden, Lloyd, & Wrighton, 2016), are likely dependent on other microorganisms for basic cellular building blocks (Brown et al., 2015; Kantor et al., 2013). Other than the two occurrences of Yanofskybacteria species in deep samples close to the soil‐weathered shale transition (127 cm and 170 cm from PLM3 and PLM6, respectively), bacteria from CPR phyla were detected only in the floodplain samples. CPR bacteria are often found in anaerobic environments and have streamlined genomes, lacking many genes for independent survival. Many are likely obligate symbionts, and as such they may often associate with anaerobic hosts, although the identities of their hosts remain unclear (Brown et al., 2015; Castelle & Banfield, 2018; Hug, Baker, et al., 2016).

The abundance of genes encoding methanol dehydrogenase (*mdh1*/*mxaF*/*xoxF* in Figure A6) and the catalytic subunit of carbon monoxide dehydrogenase (*coxL* in Figure A7) were consistently lower in the groundwater‐saturated floodplain samples than in any hillslope samples or floodplain samples from above the water table. Methanol dehydrogenase is involved in aerobic oxidation of methanol (which could derive from plant biomass or oxidation of methane), whereas CO dehydrogenase is involved in aerobic oxidation of CO (possibly produced by plants as a signaling molecule). Sulfite reduction may be a second biogeochemical process that differentiates microbial communities at the floodplain from those on the hillslope, particularly in samples below the water table and immediately above it, where *dsrD*, a hallmark for the reverse *dsr* pathway is relatively abundant (Anantharaman et al., 2018). Further, genes encoding for key enzymes (*codhC* and *codhD*) in the anaerobic Wood–Ljungdahl pathway for carbon fixation, and genes for nitrogen fixation (*nifDHK*) are relatively abundant at the floodplain site, specifically below groundwater and immediate above it compared to the hillslope sites. Interestingly, these samples contained the highest abundance of genes encoding for form I and II Ribulose‐1,5‐bisphosphate carboxylase/oxygenase (RubisCO) enzymes, known to play a role microbial carbon fixation (Berg et al., 2010). These patterns support the conclusion that groundwater‐saturated regions of the watershed support largely anaerobic microbial communities. Overall, the findings indicate that floodplain site metabolic potential is depth‐stratified, with one microhabitat below the water table that is colonized by organisms with anaerobic metabolisms, a second within the zone experiencing seasonal fluctuating redox conditions, and a third closer to the surface, where communities would experience oxidizing conditions throughout most of the year. A similar stratification, with a 70 cm alternating redox zone, was observed within a sediment profile from the Rifle river riverbed (Danczak et al., 2016). As in the current work, the microbial community of the alternating redox zone is easily distinguishable from those in both the shallow and deep zones. Overall, the spatial layout of the compartments may support complete redox cycles, analogous to sulfur cycling at oxygen‐minimum zones in the ocean (Canfield et al., 2010).

Selenium concentration may be a major factor that differentiates microbial communities at the floodplain from those on the hillslope. Selenium occurs in insoluble metal selenides in Mancos Shale that underlies much of the Gunnison River basin (Colorado, USA; Elrashidi, 2018), which includes the East River watershed. Oxidation of selenium to soluble selenite and selenate under mildly reducing to oxidizing conditions (Presser, 1994) leads to its mobilization and probably accounts for its presence in pore fluids. Enrichment of *srdA* genes, which encode the catalytic subunit of the complex required for selenate reduction, in sequences from the floodplain site suggests that dissimilatory reduction of selenate (Fakra et al., 2015; Ike, Takahashi, Fujita, Kashiwa, & Fujita, 2000; Maiers, Wichlacz, Thompson, & Bruhn, 1988; Nancharaiah & Lens, 2015; Williams et al., 2013) supports microbial growth at this site. *Geobacter* species, which were identified almost exclusively in floodplain samples (Figure A4, clade 3) and are sometimes capable of selenite reduction (Pearce et al., 2009), may be responsible for these reactions. The detection of *srdA* genes in the three deepest samples from the hillslope (127–200 cm) suggests that selenate reduction may occur periodically close to the weathered shale–soil interface where seasonally variable redox conditions induced by groundwater fluctuations may enable microbe‐catalyzed selenium transformations.

Across the hillslope sites, shallow soils have relatively similar community compositions. This might be explained by the low soil moisture that these locations experience over much of the year, as well as exposure to low temperatures during late fall and early winter prior to the onset of insulating snow cover. Further, soil community compositions are homogenized at some sites, likely due to soil mixing as a result of gopher activity (Yoo, Amundson, Heimsath, & Dietrich, 2005). Bioturbation may increase soil porosity and permeability and homogenize the mineral matrix and microbial community composition within a site, particularly close to the soil surface (reviewed by Platt, Kolb, Kunhardt, Milo, & New, 2016). It is also possible that similarity in vegetation at the nonfloodplain sites contributes to community similarity.

Between‐site heterogeneity, which could arise due to periodic events or local changes in vegetation, could be eliminated by microbial dispersal. However, microbial dispersal is generally very limited in soils that are not saturated with groundwater (Elsas, Trevors, & Overbeek, 1991). Although groundwater and runoff from rain and snowmelt might transport microbes downslope and into the weathered rock, hydraulic measurements show that overland and lateral underground transport is likely limited at the hillslope sites (T. K. Tokunaga, J. Wan, K. H. Williams, W. Brown, A. Henderson, Y. Kim, A. P. Tran, M. E. Conrad, M. Bill, R. W. H. Carroll, W. Dong, Z. Xu, A. Lavy, B. Gilbert, S. Romero, J. N. Christensen, B. Faybishenko, B. Arora, E. R. Siirila‐Woodburn, R. Versteeg, J. H. Raberg, J. E. Peterson, & S. S. Hubbard, Unpublished data). Soil and weathered rock are water‐saturated for only a few weeks each year, other than at the floodplain. During this period, water moves at ~ 10 to 20 m per month parallel to the surface slope (Tokunaga et al., under review), distances that are too short to connect communities at our sampling sites.

Our study of a hillslope lower montane meadow to floodplain transect revealed an ecosystem comprised of distinct subsystems. Specifically, our results documenting the abundance patterns of genes involved in selenium, sulfur, carbon, and nitrogen cycles suggest that hillslope and floodplain sites constitute distinct ecosystem compartments. Further, the hillslope sites are spatially differentiated into microhabitats close to (or within) weathered shale and proximal to the surface. Similarly, the floodplain site is resolved into largely anaerobic and aerobic communities over relatively short vertical distance, raising the possibility of elemental cycling across the interface. These results clarify the scale of heterogeneity in biogeochemical processes and improve our understanding of how these processes map onto the watershed.

The ability to make predictions at more than one level of resolution requires identification of the processes of interest and the parameters that affect these processes at different scales (Turner, Dale, & Gardner, 1989). For that purpose, the current work focuses on the centimeter to meters scale, serving as a starting point for a “bottom‐up” approach for exploring microbial ecology across the watershed.

The microhabitats that were identified in the hillslope and floodplain compartments of the watershed may be considered as “systems within systems” at a local scale. However, the term might also be applicable at a larger scale—one that spans across the entire watershed. Once validated by sampling at other hillslope and floodplain locations across the watershed, extrapolation of this knowledge could be used to improve our understanding of ecosystem functioning.

## CONFLICT OF INTERESTS

The authors declare no competing interests in this study.

## AUTHOR CONTRIBUTION

A.L designed research, performed research, analyzed data, and wrote the paper. D.G.M assisted in field and laboratory work. P.B.M.C conducted fieldwork. J.W performed chemistry analysis. Assisted in designing and conducting fieldwork. T.K.T conducted hydrological measurements. Assisted in designing and conducting fieldwork. B.C.T provided computational infrastructure and written bioinformatical software used in this work. K.H.W took part in the research design. Assisted in fieldwork. S.S.H took part in the research design. J.F.B supervised the study and mentored the first author.

## Data Availability

Raw reads are available through the NCBI Short Reads Archive. Accession number for each sample is provided in Table A2. Other datasets are available at: Amino acid sequences of *rpS3* genes: https://doi.org/10.6084/m9.figshare.8030792.v1. Amino acid sequences of key metabolic enzymes: https://doi.org/10.6084/m9.figshare.8030762. HMMs used in the current study: https://doi.org/10.6084/m9.figshare.8030714.v1. Phylogenetic tree of rpS3 genes: https://doi.org/10.6084/m9.figshare.8041352.

## APPENDIX A

**Table A1:** Description of Hidden Markov Models (HMMs) and their cutoffs

Metabolism	General function	Gene symbol	Gene name/function	Origin	HMM file name	Cutoff score type	Cutoff score	E‐value cutoff	Length cutoff (aa)
Aerobic respiration	Oxygen as electron acceptor	qoxA	Cytochrome aa3 quinol oxidase, subunit II	TIGRFAM	TIGR01432	NC built‐in			
Aerobic respiration	Oxygen as electron acceptor	qoxB	Cytochrome aa3 quinol oxidase, subunit I	TIGRFAM	TIGR02882	NC built‐in			
Aerobic respiration	Oxygen as electron acceptor	cydA	Cytochrome bd terminal oxidase subunit I	PFAM	PF01654	Domain	25	1.00E‐20	210
Aerobic respiration	Oxygen as electron acceptor	cydB	Cytochrome d ubiquinol oxidase, subunit II	TIGRFAM	TIGR00203	NC built‐in			
Aerobic respiration	Oxygen as electron acceptor	coxA	Cytochrome c oxidase, subunit I	TIGRFAM	TIGR02891	NC built‐in			
Aerobic respiration	Oxygen as electron acceptor	coxB	Cytochrome c oxidase, subunit II	TIGRFAM	TIGR02866	NC built‐in			
Aerobic respiration	Oxygen as electron acceptor	ccoN	Cytochrome c oxidase, cbb3‐type, subunit I	TIGRFAM	TIGR00780	NC built‐in			
Aerobic respiration	Oxygen as electron acceptor	ccoO	Cytochrome c oxidase, cbb3‐type, subunit II	TIGRFAM	TIGR00781	NC built‐in			
Aerobic respiration	Oxygen as electron acceptor	ccoP	Cytochrome c oxidase, cbb3‐type, subunit III	TIGRFAM	TIGR00782	NC built‐in			
Aerobic respiration	Oxygen as electron acceptor	cyoA	Ubiquinol oxidase, subunit II	TIGRFAM	TIGR01433	NC built‐in			
Aerobic respiration	Oxygen as electron acceptor	cyoD	Cytochrome o ubiquinol oxidase subunit IV	TIGRFAM	TIGR02847	NC built‐in			
Aerobic respiration	Oxidation of CO to CO2 under aerobic conditions	coxL	Carbon‐monoxide dehydrogenase, large subunit	TIGRFAM	TIGR02416	NC built‐in			
Aerobic respiration	Oxidation of CO to CO2 under aerobic conditions	coxM	Carbon‐monoxide dehydrogenase, medium subunit	Custom (Anantharaman et al. 2018)	carbon_monoxide_dehydrogenase_coxM	Total	184	1.00E‐20	150
Aerobic respiration	Oxidation of CO to CO2 under aerobic conditions	coxS	Carbon‐monoxide dehydrogenase, small subunit	Custom (Anantharaman et al. 2018)	carbon_monoxide_dehydrogenase_coxS	Total	130	1.00E‐20	80
C1 compounds	Formaldehyde oxidation	fae‐hps	Formaldehyde‐activating enzyme	TIGRFAM	TIGR03126	NC built‐in			
C1 compounds	Formaldehyde oxidation	mch	Methenyltetrahydromethanopterin cyclohydrolase	TIGRFAM	TIGR03120	NC built‐in			
C1 compounds	Formaldehyde oxidation	frmS	S‐(hydroxymethyl)glutathione dehydrogenase	TIGRFAM	TIGR02818	NC built‐in			
C1 compounds	Formaldehyde oxidation	S‐(hydroxymethyl)mycothiol dehydrogenase	S‐(hydroxymethyl)mycothiol dehydrogenase	TIGRFAM	TIGR03451	NC built‐in			
C1 compounds	Formaldehyde oxidation	fghA	S‐formylglutathione hydrolase	TIGRFAM	TIGR02821	NC built‐in			
C1 compounds	Mathanol oxidation	mdo	NDMA‐dependent methanol dehydrogenase	TIGRFAM	TIGR04266	NC built‐in			
C1 compounds	Methane metabolism	mauA	Methylamine dehydrogenase light chain	Kofam	K15228	Total	86.96		
C1 compounds	Methane metabolism	mauB	Methylamine dehydrogenase heavy chain	Kofam	K15229	Total	271.93		
C1 compounds	Methane metabolism	qhpA	Quinohemoprotein amine dehydrogenase	Kofam	K08685	Total	435.83		
C1 compounds	Methane oxidation, methanotroph	mdh1_mxaF	Methanol dehydrogenase Pyrroloquinoline quinone	Custom (Anantharaman et al. 2018)	methanol_dehydrogenase_pqq_xoxF_mxaF	Domain	550	1.00E‐20	300
C1 compounds	Methane oxidation, methanotroph	mmoB	Methane monooxygenase regulatory protein B	PFAM	PF02406	Total	22	1.00E‐20	80
C1 compounds	Methane oxidation, methanotroph	mmoD	Soluble methane monooxygenase‐binding protein MmoD	TIGRFAM	TIGR04550	NC built‐in			
C1 compounds	Methanogenesis	mcrA	Methyl‐coenzyme M reductase, alpha subunit	TIGRFAM	TIGR03256	NC built‐in			
C1 compounds	Methanogenesis	mcrB	Methyl‐coenzyme M reductase, beta subunit	TIGRFAM	TIGR03257	NC built‐in			
C1 compounds	Methanogenesis	mcrG	Methyl‐coenzyme M reductase, gamma subunit	TIGRFAM	TIGR03259	NC built‐in			
C1 compounds	Methanogenesis CO2	fhcD	Formylmethanofuran‐‐tetrahydromethanopterin N‐formyltransferase	TIGRFAM	TIGR03119	NC built‐in			
Carbon fixation	3HP‐4HB	abfD	4‐hydroxybutyryl‐CoA‐dehydratase	Custom (Anantharaman et al. 2018)	Four‐hydroxybutyryl‐CoA‐dehydratase			1.00E‐20	280
Carbon fixation	3HP‐4HB	4‐hydroxybutyryl‐CoA‐synthetase	4‐hydroxybutyryl‐CoA‐synthetase	Kofam	K18593	Total	1233.23		
Carbon fixation	3HP/3HP‐4HB	propionyl‐CoA‐synthase	Propionyl‐CoA‐synthase	Kofam	K15018	Total	1311.9		
Carbon fixation	Calvin non‐phototrophic	rubisco form I	Rubisco form I	Custom (Anantharaman et al. 2018)	rubisco_form_I	Total	500	1.00E−20	220
Carbon fixation	Calvin non‐phototrophic	rubisco form II	Rubisco form II	Custom (Anantharaman et al. 2018)	rubisco_form_II	Total	500	1.00E−20	220
Carbon fixation	Reductive TCA	aclA	ATP citrate lyase A	Custom (Anantharaman et al. 2018)	ATP_citrate_lyase_aclA	Total	215	1.00E−20	300
Carbon fixation	Reductive TCA	aclB	ATP citrate lyase B	Custom (Anantharaman et al. 2018)	ATP_citrate_lyase_aclB	Total	177	1.00E−20	200
Carbon fixation	Wood‐Ljungdahl pathway	codhC	CO dehydrogenase/acetyl‐CoA synthase, beta subunit	Kofam	K14138	Total	930.4		
Carbon fixation	Wood‐Ljungdahl pathway	codhD	CO dehydrogenase/acetyl‐CoA synthase, delta subunit	TIGRFAM	TIGR00381	NC built‐in			
Carbon fixation	Wood‐Ljungdahl pathway	fdhA	Formate dehydrogenase, alpha subunit	TIGRFAM	TIGR01591	NC built‐in			
Carbon fixation	Wood‐Ljungdahl pathway	fdhB	Formate dehydrogenase, beta subunit	TIGRFAM	TIGR01582	NC built‐in			
Iron	Metal (Iron/Manganese) oxidation/reduction	mtrC	Decaheme c‐type cytochrome	TIGRFAM	TIGR03507	NC built‐in			
Iron	Metal (Iron/Manganese) oxidation/reduction	mtrB	Decaheme c‐type cytochrome	TIGRFAM	TIGR03509	NC built‐in			
Nitrogen	Anammox	hzo	Hydrazine oxidase	Custom (Anantharaman et al. 2018)	hydrazine_oxidase_hzoA	Total	325	1.00E−20	160
Nitrogen	Anammox	hzs	Hydrazine synthase	Custom (Anantharaman et al. 2018)	hydrazine_synthase_hzsA	Total	466	1.00E−20	400
Nitrogen	Anammox	nirS	Nitrite reductase	Custom (Anantharaman et al. 2018)	nitrite_reductase_nirS	Domain	200	1.00E−20	280
Nitrogen	Denitrification	nirK	Nitrite reductase, copper‐containing	TIGRFAM	TIGR02376	NC built‐in			
Nitrogen	Denitrification	norB	Nitric oxide reductase subunit B	Custom (Anantharaman et al. 2018)	nitric_oxide_reductase_norB	Total	80	1.00E−20	230
Nitrogen	Denitrification	norC	Nitric oxide reductase subunit C	Custom (Anantharaman et al. 2018)	nitric_oxide_reductase_norC	Domain	50	1.00E−20	75
Nitrogen	Denitrification	nosZ	Nitrous‐oxide reductase, Sec‐dependent	TIGRFAM	TIGR04246	NC built‐in			
Nitrogen	Dissimilatory nitrate reduction	napA	Periplasmic nitrate reductase, large subunit	TIGRFAM	TIGR01706	NC built‐in			
Nitrogen	Dissimilatory nitrate reduction	napB	Periplasmic nitrate reductase, diheme cytochrome c subunit	PFAM	PF03892	Total	25	1.00E−20	80
Nitrogen	Dissimilatory nitrate reduction	narG	Nitrate reductase, alpha subunit	TIGRFAM	TIGR01580	NC built‐in			
Nitrogen	Dissimilatory nitrate reduction	narH	Nitrate reductase, beta subunit	TIGRFAM	TIGR01660	NC built‐in			
Nitrogen	Dissimilatory nitrate reduction	nirB	Nitrite reductase [NAD(P)H], large subunit	TIGRFAM	TIGR02374	NC built‐in			
Nitrogen	Dissimilatory nitrate reduction	nirD	Nitrite reductase [NAD(P)H], small subunit	TIGRFAM	TIGR02378	NC built‐in			
Nitrogen	Dissimilatory nitrate reduction	nrfH	Cytochrome c nitrite reductase, small subunit	TIGRFAM	TIGR03153	NC built‐in			
Nitrogen	Nitrification	pmoA‐amoA	Methane/ammonia monooxygenase subunit A	Kofam	K10944	Total	192.77		
Nitrogen	Nitrification	pmoB‐amoB	Methane/ammonia monooxygenase subunit B	Kofam	K10945	Total	161.43		
Nitrogen	Nitrification	pmoC‐amoC	Methane/ammonia monooxygenase subunit C	Kofam	K10946	Total	152.7		
Nitrogen	Nitrification‐comammox	nxrA	Nitrite oxidoreductase alpha subunit	Custom (Anantharaman et al. 2018)	nitrite_oxidoreductase_nxrA	Total	350	1.00E−20	500
Nitrogen	Nitrification‐comammox	nxrB	Nitrite oxidoreductase beta subunit	Custom (Anantharaman et al. 2018)	nitrite_oxidoreductase_nxrB	Domain	250	1.00E−20	200
Nitrogen	Nitrogen Fixation	nifD	Nitrogenase molybdenum‐iron protein alpha chain	TIGRFAM	TIGR01282	NC built‐in			
Nitrogen	Nitrogen Fixation	nifH	Nitrogenase iron protein	TIGRFAM	TIGR01287	NC built‐in			
Nitrogen	Nitrogen Fixation	nifK	Nitrogenase molybdenum‐iron protein beta chain	TIGRFAM	TIGR01286	NC built‐in			
Nitrogen	Nitrous oxide reduction	nosD	Nitrous oxide reductase family maturation protein	TIGRFAM	TIGR04247	NC built‐in			
Sulfur	Dissimilatory sulfate reduction and sulfide oxidation	aprA	Adenylylsulfate reductase, alpha subunit	TIGRFAM	TIGR02061	NC built‐in			
Sulfur	Dissimilatory sulfate reduction and sulfide oxidation	dsrA	Sulfite reductase alpha subunit	TIGRFAM	TIGR02064	NC built‐in			
Sulfur	Dissimilatory sulfate reduction and sulfide oxidation	dsrB	Sulfite reductase beta subunit	TIGRFAM	TIGR02066	NC built‐in			
Sulfur	Dissimilatory sulfate reduction	dsrD	Dissimilatory sulfite reductase D	PFAM	PF08679	Total	50	1.00E−20	30
Sulfur	Dissimilatory sulfate reduction and sulfide oxidation	sat	Sulfate adenylyltransferase	TIGRFAM	TIGR00339	NC built‐in			
Sulfur	Sulfate reduction	asrA	Sulfite reductase, subunit A	TIGRFAM	TIGR02910	NC built‐in			
Sulfur	Sulfate reduction	asrB	Sulfite reductase, subunit B	TIGRFAM	TIGR02911	NC built‐in			
Sulfur	Sulfate reduction	asrC	Sulfite reductase, subunit C	TIGRFAM	TIGR02912	NC built‐in			
Sulfur	Sulfide oxidation	fcc	Flavocytochrome c sulfide de­≠hydrogenase	Kofam	K17230	Domain	89.1		
Sulfur	Sulfide oxidation	sdo	Sulfur dioxygenase	Custom (Anantharaman et al. 2018)	sulfur_dioxygenase_sdo	Total	170	1.00E−20	110
Sulfur	Sulfide oxidation	sqr	Sulfide quinone oxidoreductase	Custom (Anantharaman et al. 2018)	sulfide_quinone_oxidoreductase_sqr	Total	270	1.00E−20	200
Sulfur	Thiosulfate Oxidation	soxB	Thiosulfohydrolase SoxB	TIGRFAM	TIGR04486	NC built‐in			
Sulfur	Thiosulfate Oxidation	soxC	Sulfite dehydrogenase	TIGRFAM	TIGR04555	NC built‐in			
Sulfur	Thiosulfate Oxidation	soxD	S‐disulfanyl‐L‐cysteine oxidoreductase	Kofam	K22622	Domain	133.73		
Sulfur	Thiosulfate Oxidation	soxY	Ahiosulfate oxidation carrier protein SoxY	TIGRFAM	TIGR04488	NC built‐in			
Sulfur	Thiosulfate reduction	phsA	Ahiosulfate reductase / polysulfide reductase chain A	Kofam	K08352	Total	516.13		
Arsenic	Arsenite oxidation	aoxA	Arsenite oxidase, small subunit	TIGRFAM	TIGR02694	NC built‐in			
Arsenic	Arsenite oxidation	aoxB	Arsenite oxidase, large subunit	TIGRFAM	TIGR02693	NC built‐in			
Arsenic	Arsenite reduction	arsC( glutathione/glutaredoxin type)	Arsenate reductase, glutathione/glutaredoxin type, arsC	TIGRFAM	TIGR02689	NC built‐in			
Arsenic	Arsenite reduction	arsC (glutaredoxin)	ArsC (glutaredoxin)	TIGRFAM	TIGR00014	NC built‐in			
Selenate	Selenate reduction	srdA	Selenate reductase subunit A	Custom	srdA	Total	768	1.00E−20	500

**Table A2:** Sequencing depth and assembly information

Site	Sample name	Depth (cm)	Extraction	Sequencing depth (Gbp)	# Reads	% Reads mapped	Longest scaffold (bp)	# Scaffolds longer than 1 Kbp	SRA accession #
PLM0	PLM0_5_b1	5	DNA only	12.306	85449048	8.0	34,767	39,307	SRX3939289
	PLM0_5_coex	5	Co‐extracted	14.129	96943332	8.6	30,949	51,831	SRX3938852
	PLM0_30_b1	30	DNA only	12.362	85166310	20.6	134,7726	93,948	SRX3939244
	PLM0_30_coex	30	Co‐extracted	12.272	84240060	23.1	257,168	111,705	SRX3938851
	PLM0_60_b1	60	DNA only	12.710	87758664	23.7	562,722	113,728	SRX3939286
	PLM0_60_coex_redo	60	Co‐extracted	25.024	169417374	39.0	272,482	327,331	SRX4020904
PLM1	PLM1_5_b1	5	DNA only	14.318	98155074	9.2	153,312	51,156	SRX3939403
	PLM1_5_coex_redo	5	Co‐extracted	27.169	184002508	24.7	216,442	207,336	SRX4020906
	PLM1_30_b1	30	DNA only	10.811	75007806	16.0	218,091	63,823	SRX3939421
	PLM1_30_coex	30	Co‐extracted	12.997	89479460	21.3	198,543	104,859	SRX3938854
	PLM1_60_b1_redo	60	DNA only	19.132	130188644	43.2	453,003	250,231	SRX4020708
	PLM1_60_coex	60	Co‐extracted	12.583	86855340	24.9	237,742	117,222	SRX3939072
	PLM1_100_b1	100	DNA only	11.054	75705250	33.2	345,507	120,315	SRX3939422
	PLM1_100_coex	100	Co‐extracted	7.873	55923188	19.6	254,706	55,119	SRX3938897
PLM2	PLM2_5_coex	5	Co‐extracted	13.408	91377052	23.6	84,681	104,106	SRX4038478
	PLM2_5_b1	5	DNA only	47.133	319389276	40.4	641,460	625,826	SRX4394284
	PLM2_30_coex	30	Co‐extracted	21.218	142829262	39.0	139,269	266,109	SRX4394281
	PLM2_30_b1	30	DNA only	17.856	120335700	31.3	103,781	186,106	SRX4394282
PLM3	PLM3_5_b1	5	DNA only	11.627	80171890	21.8	307,370	108,605	SRX3939400
	PLM3_5_coex	5	Co‐extracted	8.035	56424100	18.6	108,695	70,402	SRX3938970
	PLM3_30_b1	30	DNA only	12.868	88583680	31.5	266,935	155,033	SRX3939453
	PLM3_30_b2	30	DNA only	9.428	65955682	23.6	139,835	90,355	SRX3939695
	PLM3_60_b1	60	DNA only	9.619	66769884	27.6	137,172	101,753	SRX3939332
	PLM3_60_coex	60	Co‐extracted	12.305	84290508	34.8	326,827	147,715	SRX3938971
	PLM3_127_b1	127	DNA only	11.380	78421338	41.0	452,947	186,029	SRX3939455
	PLM3_127_b2	127	DNA only	13.913	95493198	43.5	495,994	200,738	SRX3939725
PLM6	PLM3_1_50_b1	50	DNA only	10.630	72989082	28.4	393,885	104,399	SRX3939694
	PLM3_1_50_b2	50	DNA only	8.019	56421590	25.0	168,549	65,789	SRX3939727
	PLM3_1_170_b1	170	DNA only	12.001	82340664	34.0	304,240	147,944	SRX3939697
	PLM3_1_170_b2	170	DNA only	12.814	88076032	34.5	1,153,492	16,1473	SRX3939726
	PLM3_1_200_b1	200	DNA only	12.002	82318922	24.2	168,592	116,565	SRX3939696
	PLM3_1_200_b2	200	DNA only	11.717	80794088	25.0	168,582	113,002	SRX3939728
PLM4	PLM4_5_b1	5	DNA only	14.338	98320688	25.2	396,580	145,893	SRX3939604
	PLM4_5_coex_redo	5	Co‐extracted	22.558	152973358	40.9	319,999	347,329	SRX4020905
	PLM4_32_b1	32	DNA only	14.160	97106782	25.3	276,908	153,216	SRX3939582
	PLM4_32_coex_redo	32	Co‐extracted	23.597	160573104	39.5	126,662	336,253	SRX4020878
	PLM4_65_b1_redo	65	DNA only	26.281	178710136	59.0	298,824	481,900	SRX4020689
	PLM4_65_coex	65	Co‐extracted	12.362	84852764	42.1	350,418	172,891	SRX3938984
	PLM4_90_b1	90	DNA only	13.724	93860710	16.0	193,382	89,201	SRX3939618
	PLM4_90_b2	90	DNA only	10.573	72869638	16.1	239,238	68,870	SRX3939724
	PLM4_90_coex	90	Co‐extracted	12.319	84864034	14.0	186,768	74,487	SRX3939033

**Table A3:** Soil texture

Site	Depth (cm)	Sand (%)	Silt (%)	Clay (%)
PLM0	5	36.8	40	23.2
	10	24.8	52.1	23.2
	20	32.8	49	18.2
	30	41.6	39.7	18.8
	43	50.3	29.7	20
	50	41.3	38.1	20.6
	60	47.5	33	19.6
	69	45.7	32.5	21.8
	75	30.6	50.4	19
	85	24.4	48	27.6
PLM1	5	31.1	53.4	15.5
	15	33.6	51.7	14.8
	24	33.1	49	17.9
	34	39.1	45.7	15.2
	44	40.2	47.8	12
	53	43.6	41.9	14.5
	64	25.2	51.7	23.1
	75	24.8	52.4	22.8
	85	24.8	50	25.2
	95	35.1	42	22.8
PLM2	5	22.5	54.2	23.3
	15	22.2	52.4	25.4
	25	27.9	50.4	21.7
	35	26.3	52.7	21
	45	24.2	47.3	28.5
	85	8	46.2	45.8
PLM3	5	7.3	59.6	33.1
	15	2	74	24
	25	8.7	63.6	27.7
	35	13.3	58.5	28.2
	45	15.7	57.2	27.1
	55	14.8	54.2	30.9
	65	11.5	51.8	36.7
	75	21.4	48.2	30.3
	85	16.7	52.7	30.5
	95	26.4	46.5	27.1
	105	24.5	49.5	26
	115	23.1	51.4	25.5
	123	31.5	47.5	20.9
PLM4	5	0	55	45
	15	0.8	56.5	42.7
	25	0	62	38
	35	0.1	63.5	36.4
	45	2.5	62.1	35.3
	55	0.5	55.3	44.2
	65	10.7	49.2	40.1
	75	42.3	37.1	20.6

**Table A4:** Soil chemistry data

			PLM0			PLM1				PLM2		PLM3				PLM4			
	ID		0.1	0.3	0.685	0.05	0.34	0.64	1.03	0.05	0.35	0.05	0.35	0.65	1.31	0.05	0.35	0.65	0.95
	Depth range, cm		5–15	25–35	64–73	0–10	28–40	58–70	105–110	0–10	30–40	0–10	30–40	60–70	127–134	0–10	30–40	60–70	90–100
	Moisture (%)		12.56	10.28	10.86	28.99	16.94	15.89	10.73	32.09	14.2	44.05	24.76	20.66	18.45	63.29	35.38	53.31	saturated
	pH		6.38	6.72	6.91	7.48	6.93	7.04	7.41	7.09	7.08	7.06	7.26	7.42	7.98	7.31	7.48	7.63	7.36
TIC/TOC	IC	mg/L	98.99	49.52	18.20	144.40	47.24	36.86	20.62	44.31	48.60	51.87	29.94	24.82	25.65	102.40	24.76	34.89	49.55
	OC	mg/L	116.50	3.74	10.29	114.50	51.46	21.91	8.64	28.14	45.76	35.79	15.97	12.62	3.26	29.34	7.20	5.46	9.60
	TN	μg/L	10620.00	143.90	766.50	12930.00	6793.00	11345.00	719.50	2455.00	3001.00	2326.00	2292.00	1626.50	944.54	2140.61	3452.34	1497.54	935.52
ICP‐MS	Li	ppb	3.30	1.18	1.30	3.97	3.25	3.63	3.14	0.58	1.01	5.54	4.32	4.09	5.14	2.95	1.71	1.88	5.32
	Stdev		0.47	0.36	0.78	0.41	0.57	0.84	0.27	0.61	0.33	0.40	0.58	0.91	0.45	0.43	0.77	0.66	0.56
	B	ppb	102.42	87.61	54.83	143.34	67.02	48.00	34.96	44.61	25.02	60.76	28.12	45.81	20.83	25.63	5.46	1.23	13.42
	Stdev		2.26	2.92	1.54	2.42	2.23	1.94	1.01	1.14	0.94	0.97	1.71	1.25	1.28	1.41	0.88	1.54	1.24
	Na	ppb	1355.17	1133.33	2169.75	1816.30	1976.77	2735.24	2683.65	839.47	3111.97	2658.83	2741.90	3129.77	5154.50	6459.35	4463.97	6382.01	9178.14
	Stdev		39.32	15.95	44.56	20.73	52.60	37.43	44.59	11.78	74.58	51.19	25.62	70.14	108.66	106.31	282.17	133.33	140.39
	Mg	ppb	33411.62	12191.77	5449.18	42057.49	15046.97	16979.04	7351.49	10546.20	14864.67	15212.14	9823.32	7792.04	7064.34	25202.62	5328.35	8832.28	31457.56
	Stdev		536.25	286.94	162.25	422.76	288.02	268.02	129.02	244.60	191.04	172.16	51.02	58.58	84.68	303.73	33.87	53.27	242.35
	Al	ppb	1143.11	1488.09	638.25	593.64	2214.93	234.68	305.58	374.28	296.68	466.68	256.63	461.61	129.29	93.93	175.72	80.48	59.44
	Stdev		27.05	15.93	14.40	6.28	10.29	5.32	2.13	6.73	3.50	8.05	2.34	4.94	2.40	1.09	4.21	1.60	0.90
	Si	ppb	17253.40	11804.69	9636.01	17851.07	12967.58	7732.72	7140.03	8986.84	5724.30	9266.36	5754.88	5901.79	4709.97	8028.88	4108.63	4190.04	3793.60
	Stdev		305.91	134.93	206.30	94.38	145.83	100.90	128.97	161.90	54.55	67.46	56.66	53.38	48.33	95.10	12.59	35.91	35.60
	K	ppb	69876.52	35556.54	1730.93	176493.90	15073.27	6181.77	1809.77	24831.16	2250.39	5233.84	1080.11	851.57	3140.80	5944.42	647.13	787.93	2812.52
	Stdev		687.96	461.02	17.57	4529.23	124.07	84.33	12.12	286.68	8.29	42.57	8.39	16.09	20.24	61.57	4.85	3.29	16.96
	V	ppb	6.29	4.65	2.49	5.57	7.03	1.14	1.43	1.34	1.50	1.55	1.12	2.08	0.38	0.48	0.77	0.14	0.41
	Stdev		0.19	0.19	0.11	0.06	0.94	0.05	0.09	0.04	0.04	0.06	0.05	0.30	0.02	0.03	0.04	0.02	0.03
	Cr	ppb	2.21	1.74	1.70	1.26	2.56	0.61	0.78	0.81	0.40	0.80	0.37	1.20	1.81	0.21	0.45	0.50	0.11
	Stdev		0.27	0.07	0.11	0.04	0.38	0.06	0.11	0.04	0.02	0.03	0.03	0.20	0.06	0.02	0.04	0.05	0.02
	Mn	ppb	1075.44	132.50	125.73	692.14	94.20	10.35	15.44	199.87	111.61	64.24	3.67	6.93	1.87	2162.53	4.37	0.76	224.09
	Stdev		4.56	0.64	0.73	4.73	0.64	0.04	0.15	1.87	0.55	0.54	0.05	0.25	0.04	6.57	0.09	0.02	1.20
	Ca	ppb	74757.19	29934.74	12142.65	92501.26	35731.74	38525.57	16044.98	40618.93	55255.03	52303.10	33697.81	27905.78	25445.34	130998.75	32341.42	49413.02	328102.01
	Stdev		479.35	349.97	69.23	1988.91	274.53	407.09	149.02	312.49	357.24	503.92	127.09	153.70	127.23	1438.36	96.21	215.21	3980.56
	Fe	ppb	892.90	870.63	2594.82	430.17	1918.56	255.76	1469.97	253.59	241.18	537.94	502.27	1105.93	266.06	121.72	736.69	40.71	85.76
	Stdev		14.02	19.85	126.84	4.48	147.84	10.09	156.48	2.96	7.68	6.59	35.60	55.67	6.17	0.50	26.60	1.35	0.99
	Se	ppb	1.13	0.76	0.34	1.79	1.80	0.59	0.40	0.58	0.71	1.13	0.80	2.14	0.90	2.04	1.93	8.12	3.83
	Stdev		0.37	0.12	0.22	0.50	0.17	0.17	0.25	0.11	0.14	0.09	0.14	0.18	0.28	0.44	0.34	0.24	0.63
	Ti	ppb	24.94	17.22	13.77	16.19	24.83	6.26	7.08	7.02	5.85	10.85	5.71	7.96	4.49	4.48	4.67	3.35	3.18
	Stdev		1.05	0.44	0.65	0.53	0.36	0.45	0.32	0.32	0.32	0.53	0.32	0.40	0.40	0.21	0.31	0.27	0.20
	Ni	ppb	8.94	4.40	5.11	10.09	5.06	3.08	5.24	3.98	3.79	6.57	3.69	7.56	3.36	14.86	5.81	5.69	36.61
	Stdev		0.20	0.06	0.23	0.24	0.20	0.16	0.09	0.22	0.14	0.80	0.11	0.35	0.07	0.42	0.08	0.23	0.33
	Co	ppb	7.73	1.91	2.10	3.90	1.29	0.31	0.66	1.44	0.72	0.72	0.43	0.36	0.15	2.80	0.25	0.16	2.92
	Stdev		0.10	0.10	0.11	0.05	0.04	0.03	0.05	0.06	0.02	0.06	0.02	0.03	0.02	0.06	0.02	0.03	0.08
	Cu	ppb	12.70	6.57	7.97	11.39	12.26	4.71	8.62	4.41	3.95	14.74	4.59	11.90	6.08	7.02	16.04	8.00	12.89
	Stdev		0.15	0.12	0.10	0.31	0.37	0.19	0.33	0.11	0.16	1.55	0.17	0.17	0.13	0.28	0.33	0.22	0.18
	Zn	ppb	45.09	38.90	38.06	50.42	48.26	39.17	47.91	34.53	33.18	44.51	35.24	40.60	95.69	46.80	78.56	25.29	58.99
	Stdev		0.88	0.57	0.73	0.87	0.82	1.19	0.72	0.56	0.67	6.83	0.55	0.67	0.91	0.66	1.29	1.21	0.78
	Ge	ppb	0.02	0.04	0.04	0.00	0.06	0.02	0.02	0.02	0.02	0.02	0.01	0.04	0.01	0.01	0.03	0.02	0.07
	Stdev		0.02	0.03	0.01	0.01	0.02	0.02	0.02	0.02	0.02	0.02	0.02	0.02	0.01	0.02	0.02	0.02	0.03
	As	ppb	3.23	1.56	1.39	4.24	2.21	0.73	1.51	1.48	0.90	2.05	0.54	1.56	0.38	1.85	1.12	0.23	1.21
	Stdev		0.07	0.07	0.04	0.05	0.21	0.05	0.06	0.06	0.04	0.07	0.05	0.11	0.02	0.11	0.05	0.05	0.06
	Rb	ppb	28.41	20.28	1.06	29.66	7.60	2.62	0.32	5.03	3.48	1.18	0.39	0.68	0.44	0.99	0.49	0.27	0.94
	Stdev		0.35	0.21	0.08	0.20	0.10	0.06	0.02	0.07	0.06	0.05	0.02	0.04	0.02	0.03	0.03	0.01	0.04
	Sr	ppb	208.82	84.38	28.56	288.78	105.47	113.95	45.85	131.05	169.93	194.08	129.23	103.41	94.74	442.94	111.79	160.94	742.94
	Stdev		2.01	0.97	0.43	4.86	0.61	0.54	0.29	1.20	0.73	2.80	0.78	0.48	0.35	2.55	0.47	0.51	6.32
	Zr	ppb	1.16	0.85	1.23	0.59	1.49	0.69	0.76	0.67	0.68	0.70	0.66	0.69	0.46	0.64	0.48	0.39	0.82
	Stdev		0.22	0.07	0.23	0.05	0.10	0.04	0.04	0.01	0.03	0.03	0.03	0.03	0.07	0.03	0.02	0.01	0.04
	Mo	ppb	1.35	0.61	0.34	3.02	2.22	0.93	2.52	1.00	0.73	1.15	0.69	1.24	4.00	3.61	1.25	0.61	12.69
	Stdev		0.09	0.04	0.03	0.13	0.06	0.05	0.17	0.04	0.04	0.02	0.07	0.07	0.11	0.06	0.07	0.06	0.08
	Ag	ppb	0.22	0.10	0.23	0.24	0.29	0.22	0.38	0.02	0.11	0.04	0.01	0.89	0.06	N.D	0.11	0.06	0.01
	Stdev		0.02	0.02	0.02	0.02	0.04	0.03	0.04	0.01	0.01	0.02	0.01	0.17	0.02	N.D	0.01	0.01	0.01
	Cd	ppb	0.28	0.11	0.13	0.39	0.45	0.18	0.24	0.19	0.19	0.29	0.13	0.24	0.05	0.78	0.15	0.32	0.35
	Stdev		0.03	0.02	0.04	0.06	0.03	0.02	0.05	0.04	0.02	0.07	0.05	0.06	0.05	0.14	0.03	0.05	0.04
	Sb	ppb	0.28	0.18	0.31	0.29	0.34	0.18	0.54	0.15	0.27	0.29	0.20	0.37	0.29	0.77	0.69	0.52	4.97
	Stdev		0.04	0.01	0.03	0.03	0.05	0.02	0.06	0.03	0.04	0.06	0.02	0.01	0.04	0.03	0.03	0.03	0.11
	Cs	ppb	0.15	0.16	0.08	0.85	0.24	0.02	0.01	0.05	0.04	0.07	0.02	0.05	0.02	0.01	0.03	0.01	0.01
	Stdev		0.02	0.01	0.01	0.02	0.01	0.00	0.00	0.00	0.01	0.01	0.00	0.00	0.01	0.00	0.01	0.00	0.00
	Ba	ppb	362.84	144.17	30.64	388.07	131.29	90.98	34.53	136.47	98.59	129.90	68.57	62.34	41.35	216.84	41.74	88.78	164.14
	Stdev		1.25	0.85	1.05	3.13	0.88	0.98	0.70	1.14	1.21	1.54	0.44	1.13	0.57	1.36	0.91	0.61	1.00
	Eu	ppb	0.19	0.11	0.40	0.21	0.21	0.07	0.08	0.08	0.07	0.09	0.05	0.15	0.03	0.09	0.06	0.05	0.07
	Stdev		0.02	0.01	0.01	0.01	0.02	0.01	0.01	0.01	0.01	0.02	0.01	0.02	0.01	0.02	0.01	0.02	0.01
	Pb	ppb	1.39	1.40	6.71	3.08	3.31	0.55	2.12	0.74	0.38	1.28	0.74	1.37	0.36	0.18	1.89	0.26	0.18
	Stdev		0.04	0.05	0.13	0.20	0.14	0.10	0.08	0.03	0.03	0.47	0.04	0.02	0.02	0.03	0.06	0.02	0.01
	U	ppb	0.41	0.22	0.22	0.76	0.56	0.14	0.14	0.10	0.24	0.19	0.15	0.26	0.20	3.35	0.13	0.30	8.28
	Stdev		0.02	0.02	0.03	0.03	0.03	0.01	0.01	0.02	0.01	0.01	0.01	0.01	0.02	0.08	0.02	0.03	0.18
	P	ppb	1347.62	706.52	376.29	1965.34	918.02	433.73	355.96	438.01	445.24	554.69	334.56	381.55	285.54	408.68	262.80	340.61	4794.72
	Stdev		23.35	24.38	12.16	33.49	16.13	14.99	6.08	13.45	8.31	17.42	13.34	17.16	21.14	16.20	9.43	38.91	1246.07
IC	Sulfate	mg/L	1.71	1.31	0.90	4.60	4.13	NA	2.38	NA	NA	8.87	5.30	4.57	8.83	5.78	4.83	19.93	730.04
	Nitrate	mg/L	2.00	NA	2.31	1.18	3.55	36.12	1.03	2.25	2.06	1.66	4.06	3.18	2.43	1.77	10.49	4.74	1.10

Note

1:1 (soil:DIW mass ratio) extraction. The original porewater was taken into account as a part of the total water mass.

Uncertainty for pH measurements ±0.05.

Uncrertainty for TIC/TOC <3.

Uncrertainty for anions IC <5%.
